# Dynein Light Intermediate Chain 2 Facilitates the Metaphase to Anaphase Transition by Inactivating the Spindle Assembly Checkpoint

**DOI:** 10.1371/journal.pone.0159646

**Published:** 2016-07-21

**Authors:** Sagar P. Mahale, Amit Sharma, Sivaram V. S. Mylavarapu

**Affiliations:** 1 Laboratory of Cellular Dynamics, Regional Centre for Biotechnology, National Capital Region Biotech Science Cluster, Faridabad, Haryana, India; 2 Affiliated to Manipal University, Manipal, Karnataka, India; Florida State University, UNITED STATES

## Abstract

The multi-functional molecular motor cytoplasmic dynein performs diverse essential roles during mitosis. The mechanistic importance of the dynein Light Intermediate Chain homologs, LIC1 and LIC2 is unappreciated, especially in the context of mitosis. LIC1 and LIC2 are believed to exist in distinct cytoplasmic dynein complexes as obligate subunits. LIC1 had earlier been reported to be required for metaphase to anaphase progression by inactivating the kinetochore-microtubule attachment-sensing arm of the spindle assembly checkpoint (SAC). However, the functional importance of LIC2 during mitosis remains elusive. Here we report prominent novel roles for the LIC2 subunit of cytoplasmic dynein in regulating the spindle assembly checkpoint. LIC2 depletion in mammalian cells led to prolonged metaphase arrest in the presence of an active SAC and also to stretched kinetochores, thus implicating it in SAC inactivation. Quantitative fluorescence microscopy of SAC components revealed accumulation of both attachment- and tension-sensing checkpoint proteins at metaphase kinetochores upon LIC2 depletion. These observations support a stronger and more diverse role in checkpoint inactivation for LIC2 in comparison to its close homolog LIC1. Our study uncovers a novel functional hierarchy during mitotic checkpoint inactivation between the closely related but homologous LIC subunits of cytoplasmic dynein. These subtle functional distinctions between dynein subpopulations could be exploited to study specific aspects of the spindle assembly checkpoint, which is a key mediator of fidelity in eukaryotic cell division.

## Introduction

The fidelity of eukaryotic mitotic cell divisions is imperative for viability and function of the daughter cells, and to ensure that the two daughters contain the correct dose of chromosomes. Precise mitotic regulation is ensured through multiple pathways and events at the various stages of mitosis in the mother cell. Mis-regulation of these critical pathways or slippage through these regulatory mechanisms leads to aberrant mitosis, chromosome mis-segregation and aneuploidy, which are well-established precursors to major diseases like cancer and polycystic kidney disease [1–4]. Elucidation of the molecular mechanisms of mitotic regulation is imperative to understand the basis for asymmetric stem cell division leading to differentiation, as well as for potential therapeutic intervention in major diseases [5–9].

The major quality-control mechanism in the metaphase to anaphase cell cycle transition is the Spindle Assembly Checkpoint (SAC), mediated through SAC effector proteins that localize at kinetochores in early mitosis [10–13]. The SAC ensures that sister chromatids of all chromosomes are equally segregated in anaphase to future daughter cells by arresting cells in metaphase until its conditions—bipolar spindle attachment of all sister chromatids and subsequent inter-kinetochore tension—are satisfied. SAC proteins are subsequently removed (stripped) from kinetochores primarily by the pluripotent molecular motor cytoplasmic dynein, to achieve SAC inactivation and facilitate anaphase onset [1, 14, 15]

Cytoplasmic dynein 1 is a multisubunit protein motor complex comprising of six different subunit families [16–19]. The highly homologous Light Intermediate Chain subunits of dynein, LIC1 and LIC2 are about 65% identical in primary sequence. LIC1 and LIC2 are present in biochemically distinct dynein complexes and have been reported to play distinct functional roles in mitosis [20, 21]. Specifically, LIC1 regulates the metaphase to anaphase transition [22] and LIC2 ensures completion of cytokinesis [23], the terminal step of mitosis. However, the mechanistic distinctions between these subunits with respect to mitosis are unclear.

Light Intermediate Chain 1 (LIC1) was recently identified as the subunit of dynein that specifically mediates removal of SAC proteins from metaphase kinetochores [22, 24]. LIC1 was implicated in specifically removing the attachment-sensing subset of SAC proteins from metaphase kinetochores, without significantly affecting dynein complex assembly or the other functions of dynein [22]. Light intermediate chain 2 (LIC2) is a close homolog of LIC1 reported to influence cytokinetic progression [23]. However, the comprehensive functions of LIC2 in mitosis are not understood. The intracellular localization of LIC2 is primarily at spindle poles throughout the cell cycle [25] including during mitosis, suggesting a strong mitotic function at spindle poles. A potential additional role for LIC2 at chromosomal kinetochores in metaphase is enticing to explore given the sequence similarity with LIC1, but has not been probed in detail. A recent structure-function study illuminated the biochemical basis for assembly of LICs into the dynein complex and suggested cargo-binding mechanisms for the LICs through their C-terminal domains [26, 27]. A thorough functional dissection of the LICs however remains elusive and is necessary to understand their mitotic contributions, as well as to discern the evolutionary significance of the emergence of two LICs in vertebrates.

We report here key roles for LIC2 in regulating mitotic progression. LIC2 is required for proper metaphase to anaphase transition independent of LIC1. Using quantitative fluorescence microscopy, we demonstrate that LIC2 facilitates anaphase onset by inactivating the SAC at metaphase, causing the removal of both attachment and tension sensing SAC proteins from metaphase kinetochores. This distinguishes LIC2 from LIC1, which removes primarily the attachment sensors. Our studies demonstrate LIC2 as the dominant subunit of cytoplasmic dynein with respect to mitotic checkpoint inactivation.

## Results

### LIC2 is required for metaphase to anaphase progression

**Fig 1:**
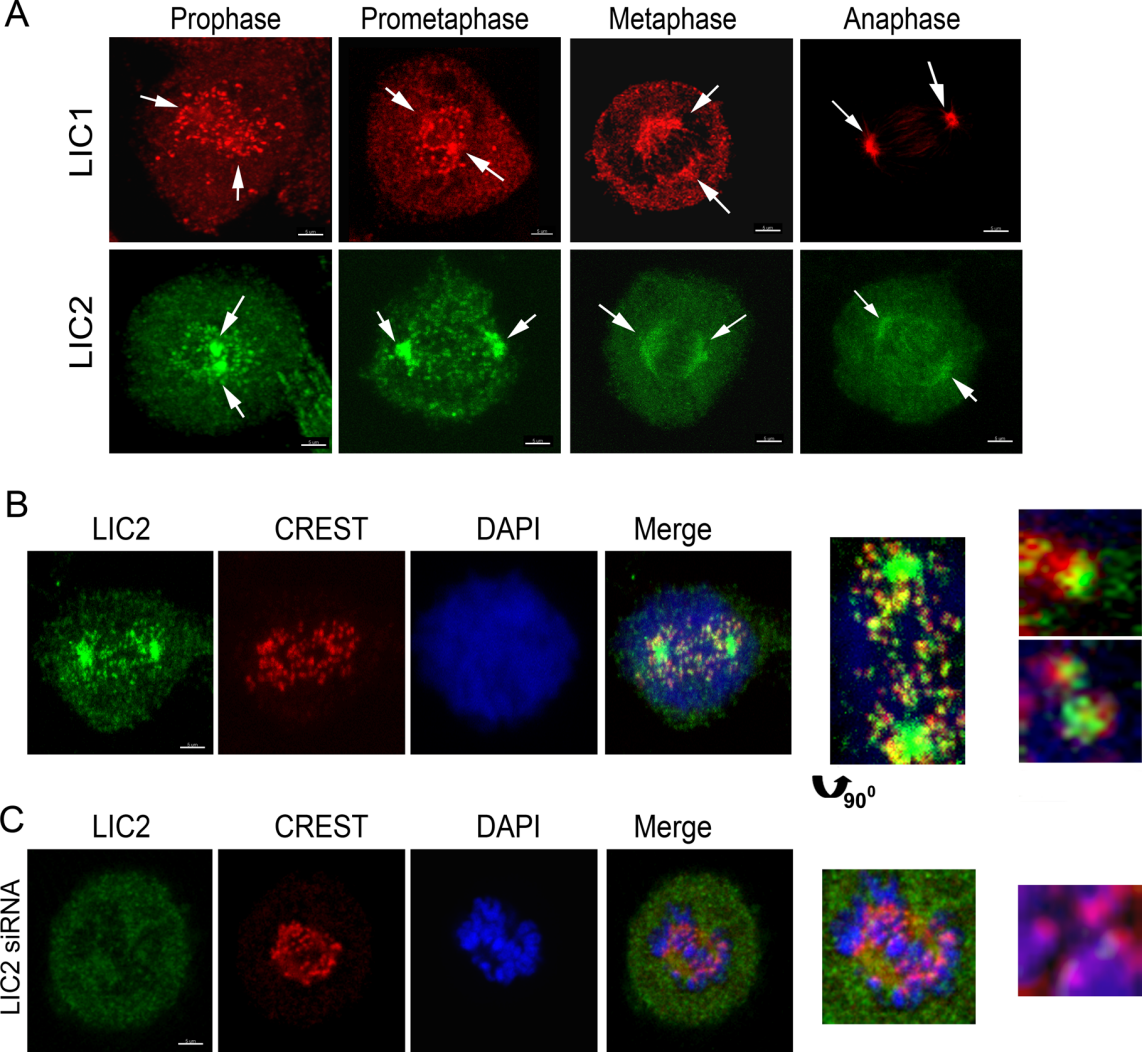
Localization of LICs in Mitosis. A) Confocal immunofluorescence images of Hela cells depicting the localization of LIC1 (red) and LIC2 (green), shown by arrows. B) Fluorescence images of Hela cells showing the presence of LIC2 (green, using the ThermoScientific antibody) at kinetochores (CREST, red) in prometaphase. C) Fluorescence imaging after treatment of cells with anti-LIC2 siRNA, showing loss of LIC2 signal from spindle poles and kinetochores (arrow in inset). DAPI was used to visualize chromosomes. Scalebar is 5 μm in all images that are not zoomed.

**Fig 2:**
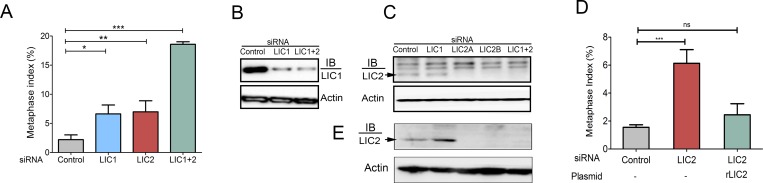
LIC2 is required for metaphase to anaphase progression. A) Metaphase index (% of total cells present in metaphase **+/-** SD) in Hela cells treated with respective siRNAs as indicated. (3 experiments, n = approximately 500 cells per experiment). B) & C) Western blots showing siRNA mediated LIC1 and LIC2 specific knockdown. LIC2a and LIC2b represent two different siRNA sequences against human LIC2 [23]. The band marked by the arrow represents human LIC2 that gets reduced upon siRNA treatment, the upper bands are non-specifically recognized by the LIC2 antibody (ThermoScientific). Actin = loading control. D) Rescue of the LIC2-depletion induced metaphase arrest by transgenic expression of rat LIC2 (3 experiments, n = at least 500 cells per experiment). E) Western blots showing specific depletion of LIC2 upon treatment with LIC2-specific siRNAs, using a different LIC2 antibody (Abcam). LIC2a and LIC2b represent two different siRNA sequences against human LIC2 [23]. The band marked by the arrow represents human LIC2 that appears at the same molecular weight as in C.

A role for LIC2 in mediating completion of cytokinesis had been earlier reported [23]. Recently, dynein LICs were reported to have roles in mitosis [28] and in maintaining spindle bipolarity [29]. However, there has been no study relating to the role of LIC2 in mediating metaphase to anaphase progression by acting on the spindle assembly checkpoint. We first ascertained the intracellular mitotic localization of LIC2 by immunofluorescence analysis in Hela cells. LIC2 prominently decorated mitotic centrosomes starting from prophase through anaphase, as also reported earlier (Fig 1A) [25, 30]. We also observed clearly discernible localization of LIC2 at kinetochores in prometaphase (Fig 1B), in addition to the localization earlier reported at mitotic spindle microtubules near the poles, as well as to the spindle midzone in anaphase (Fig 1A) [25, 30]. The localization of LIC2 at mitotic kinetochores has been reported earlier but not studied further [25, 30]. Specific LIC2 depletion using small RNA interference [23] showed an arrest in metaphase when measured in asynchronous cultures, which was independent of LIC1 (Fig 2A). We confirmed that the respective siRNAs used for the two LICs were potent to similar levels, as well as specific to their intended targets by immunoblotting (Fig 2B and 2C). Interestingly, the strength of the metaphase arrest phenotype did not increase significantly despite dosage titration or optimization of transfection conditions for introducing the LIC1/ LIC2 siRNAs, indicating saturable protein depletion (Fig 2B and 2C). When LIC2 and LIC1 were co-depleted from cells by treatment with an equimolar mixture of both siRNAs, the strength of the metaphase arrest phenotype was additive (Fig 2A), indicating at least some divergence in the mechanisms of action of the two LICs at metaphase. Exogenous expression of a rat LIC2 ortholog that was not targeted by the anti-human LIC2 siRNAs (Fig 2D, S1 Fig) rescued the metaphase arrest caused by LIC2 depletion, thus verifying the specificity of the depletion phenotype.

**Fig 3:**
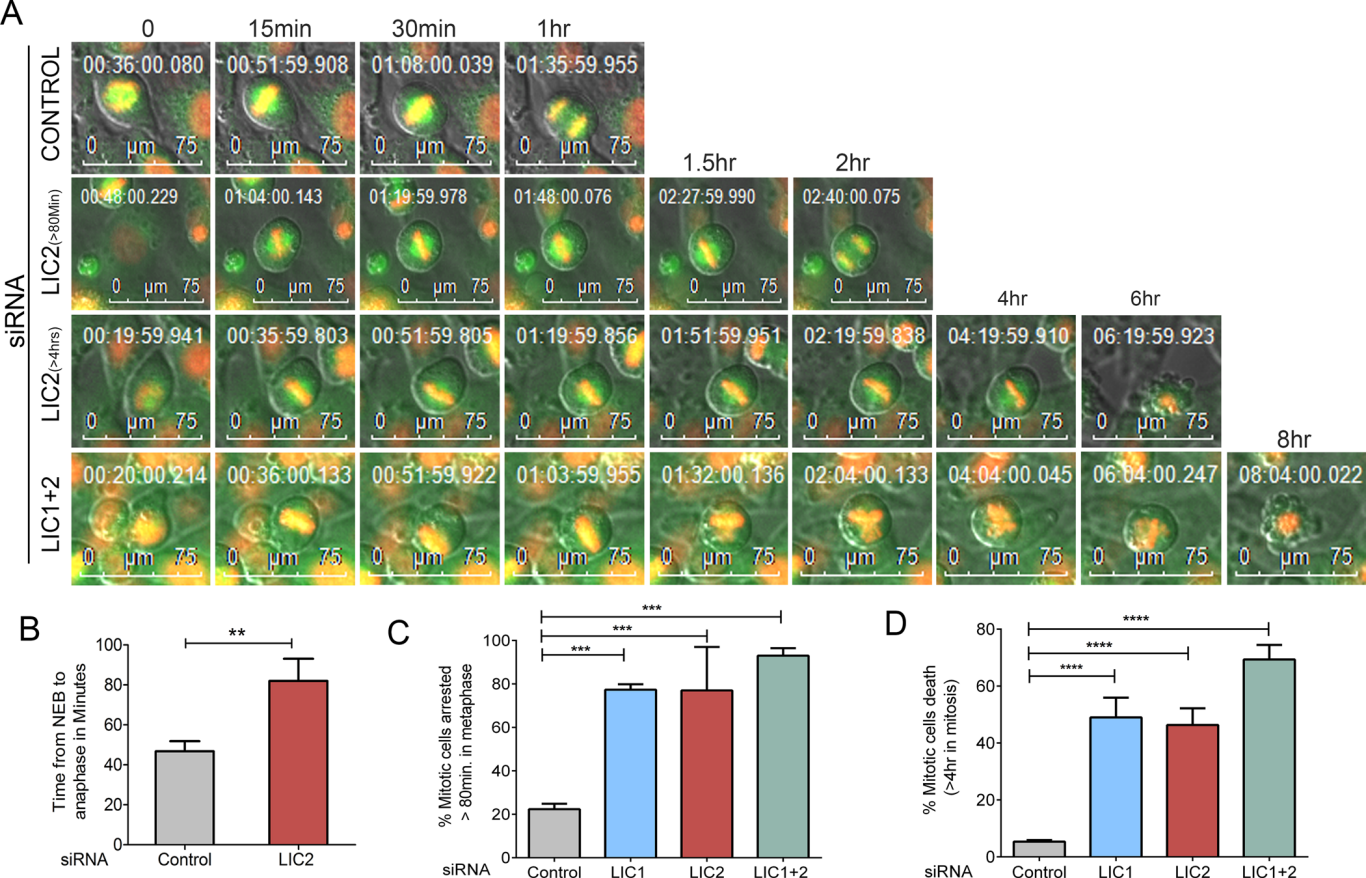
LIC2 depletion leads to prolonged metaphase arrest. A) Still images from a movie featuring four different representative siRNA treated Hela cells (control, LIC2 and LIC 1 + 2) showing prolonged arrest in metaphase, followed by delayed anaphase onset (LIC2 > 80 min) or cell death (LIC2 > 4 hrs). B) Average metaphase to anaphase timing, (3 experiments, n = at least 100 cells per experiment). NEB = Nuclear Envelope Breakdown. Statistical significance was calculated by the T-test between two groups. C) Fraction of mitotic cells arrested for more than 80 minutes in metaphase upon respective siRNA treatment. (3 experiments, n = at least 100 cells per experiment). D) Fractions of metaphase cells dying after prolomged metaphase arrest (> 4 hrs) from the cells in C. p values in all panels are * p <0.05, ** p<0.01, *** p<0.001. Scalebar is 75 μm in all images as indicated.

To further confirm the metaphase arrest, we depleted LIC2 in a Hela cell line stably expressing histone 2B-mCherry and GFP-alpha tubulin and performed time-lapse fluorescence video imaging. LIC2 depletion resulted in an increase in the average duration from nuclear envelope breakdown (NEB) to anaphase onset by about two-fold as compared to control cells (Fig 3A and 3B, S1 and S2 Movies). We further observed that upon either LIC1 or LIC2 depletion and upon co-depletion of both LICs, approximately 80% of mitotic cells stayed arrested in metaphase for abnormally extended periods (Fig 3C, minimum 80 minutes, by when most control cells had progressed to anaphase). Approximately 50% of metaphase-arrested cells enter anaphase in a delayed manner, while the remaining stay arrested in metaphase and then die (Fig 3C, S3 Movie). We observed that the majority of metaphase arrested cells depleted for both LIC1 and LIC2 underwent cell death (Fig 3A and 3D; S4 Movie).

### LIC2 acts through the spindle assembly checkpoint

**Fig 4:**
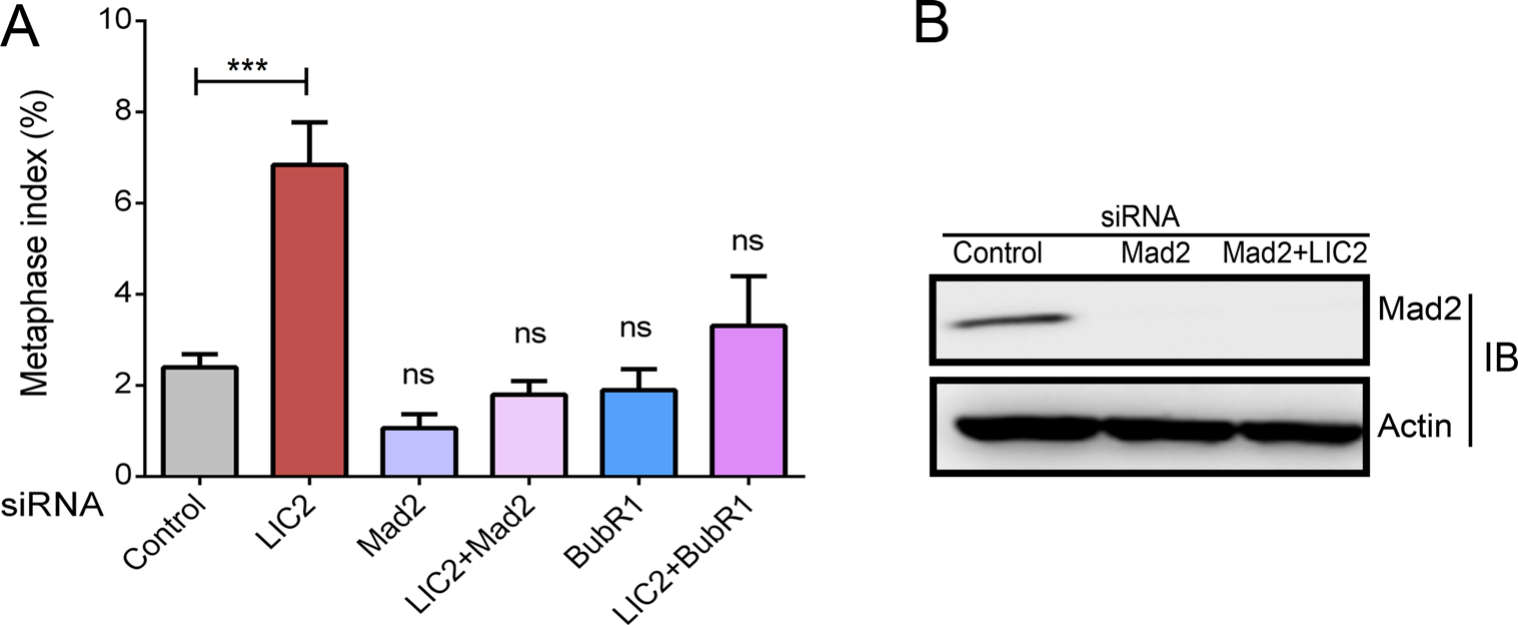
LIC2 acts on the spindle assembly checkpoint (SAC) to ensure mitotic progression. A) Metaphase index in Hela cells after respective siRNA treatments of Mad2 and BubR1 alone or co-depleted with LIC2, as indicated on the x-axis (3 experiments, n = at least 200 cells per experiment). *** p<0.001, ns = not significant (p > 0.05). B) Western blot showing depletion of Mad2 by siRNA treatment. Actin = loading control.

The potent role for LIC2 in metaphase to anaphase progression prompted us to examine whether this effect was mediated through the spindle assembly checkpoint (SAC), the major quality control mechanism governing the fidelity of chromosome segregation [10, 11]. The SAC delays anaphase onset until both its major quality-control parameters—bipolar attachment of all kinetochore pairs to kinetochore microtubules, followed by establishment of tension between sister-kinetochores due to contracting kinetochore microtubules–are satisfied. Inhibition of the SAC by depletion of the key SAC effectors Mad2 or BubR1 led to rapid progression of cells through metaphase, as expected (Fig 4A). Interestingly, co-depletion of LIC2 in combination with either Mad2 or BubR1 (Fig 4A and 4B, inactive SAC background) did not result in any metaphase arrest otherwise seen with LIC2 depletion, strongly suggesting the SAC as a major target of action of LIC2 at metaphase (Fig 4A). Thus, the metaphase arrest due to LIC2 depletion was mediated through an active SAC, like for its homolog LIC1 [22].

**Fig 5:**
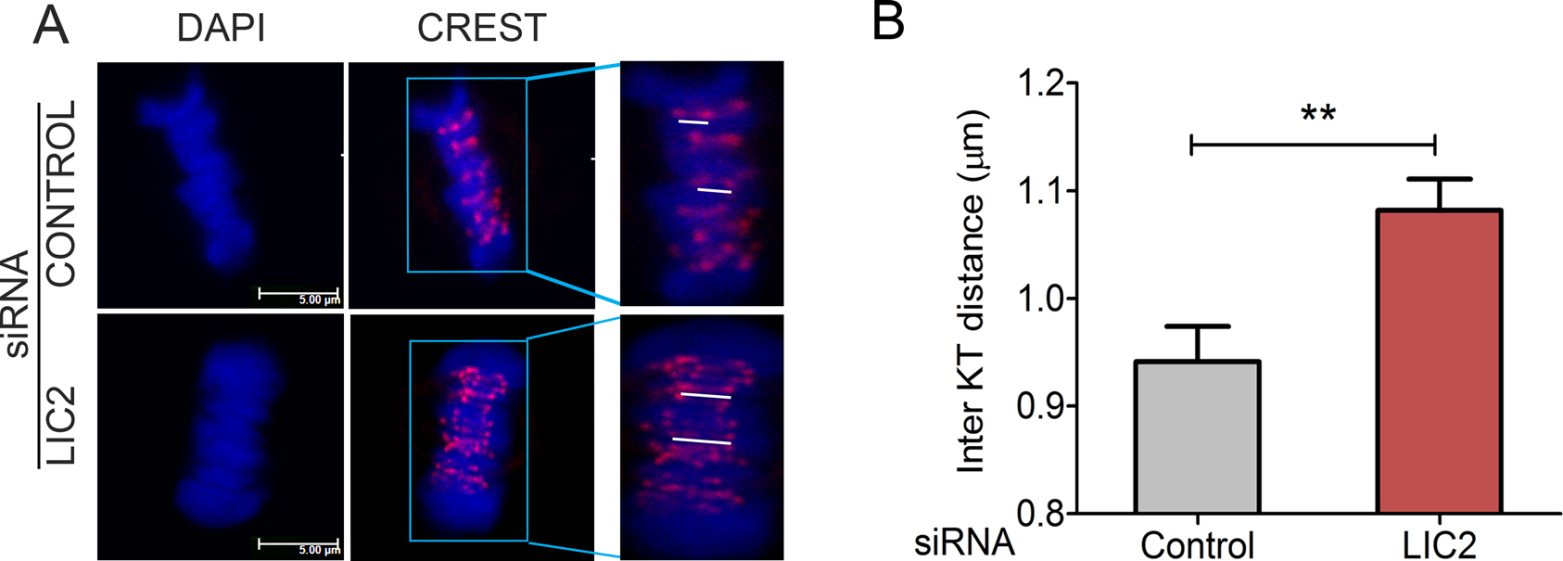
LIC2 depletion leads to increased inter-kinetochore tension in metaphase. A) Confocal immunofluorescence images of metaphase Hela cells stained for kinetochores (red, CREST) and chromatin (DAPI). White lines indicate representative distance measurements between sister kinetochores. The insets on the right are zoomed to equivalent levels in both upper and lower panels. B) Average inter-kinetochore distance (μm) on y-axis. 3 experiments; n = at least 30 metaphase cells per experiment (10 pairs of sister chromatids measured per cell). ** = p < 0.01). Scalebar is 5 μm in all images that are not zoomed.

A characteristic feature of prolonged SAC-arrested metaphase cells is the establishment of inter-kinetochore tension between sister kinetochores, which manifests as an increased inter-kinetochore distance [22, 31]. We measured the inter-kinetochore distance in well congressed metaphase cells that had been depleted of LIC2 by siRNA treatment and compared with control siRNA treated cells (Fig 5A). Indeed, LIC2 depleted metaphase cells showed higher average inter-kinetochore distances (Fig 5B), suggesting the presence of an active SAC at metaphase kinetochores despite the establishment of inter-kinetochore tension. These results indicated inefficient inactivation of the SAC at metaphase upon LIC2 depletion and together pointed towards a direct role for LIC2-dynein in regulation of the SAC at metaphase kinetochores.

### LIC2 is required for removal of spindle assembly checkpoint proteins from metaphase kinetochores

**Fig 6:**
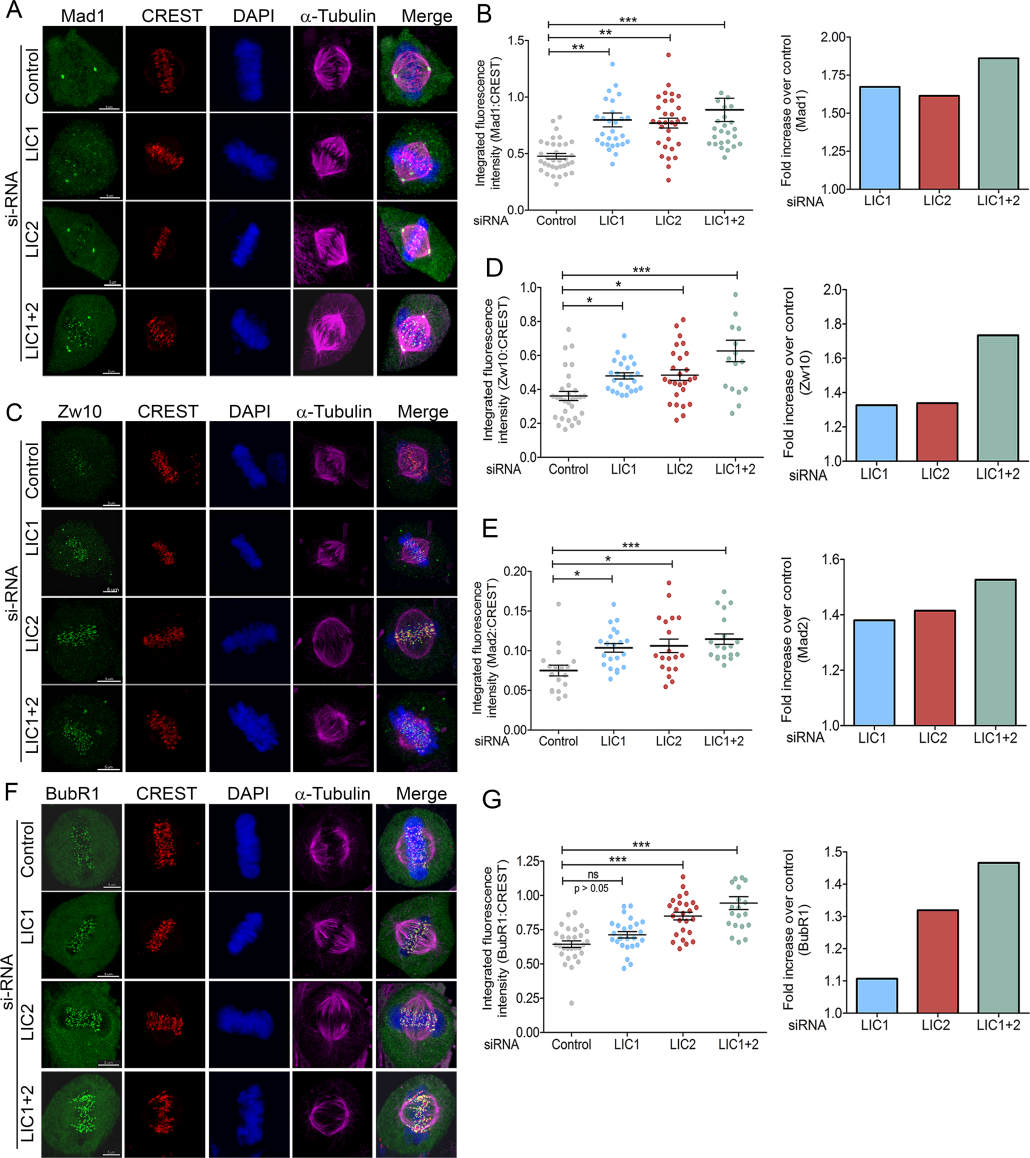
LIC2 removes key SAC proteins from metaphase kinetochores to inactivate the spindle assembly checkpoint. Representative images showing accumulation of SAC proteins A) Mad1, C) Zw10 and F) BubR1 at metaphase kinetochores upon prolonged metaphase arrest following indicated siRNA treatments. B, D, E, G) Left panels: integrated fluorescence intensities of SAC proteins normalized to the respective kinetochore (CREST) intensities for B) Mad1, D) Zw10, E) Mad2 and G) BubR1 at metaphase kinetochores. Y-axis = mean fluorescence intensity (+/- SEM) from 3 independent experiments, n = at least 18 metaphase cells per experiment for LIC1 and LIC2 depletion. Right panels: fold increase in normalized fluorescence intensity over control (GFP siRNA) for the various SAC proteins. Scalebar is 5 μm in all images.

**Fig 7:**
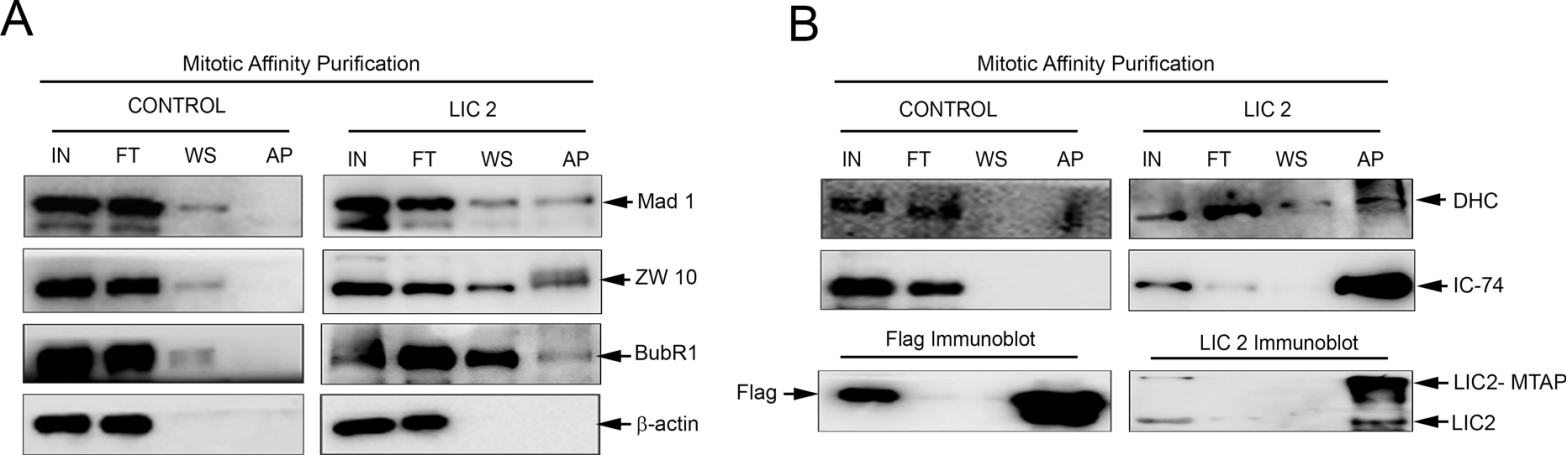
LIC2 biochemically interacts with key SAC proteins at metaphase. A) Western blots of affinity purified mitotic LIC2 (LIC2-MTAP) probing for interaction of SAC proteins Mad1, Zw10 and BubR1 (right panel), and with the empty tag control (left panel). ß-actin was used as the loading control. Control indicates empty tag (MTAP vector alone) pulldown, LIC2 indicates LIC2-MTAP (see Materials and Methods). B) Upper panels: Immunoblots probing for the pulldown of dynein subunits DHC (dynein heavy chain) and IC-74 (dynein intermediate chain) upon affinity purification of the empty MTAP tag or LIC2-MTAP. Lower panels show successful pulldown of the respective bait proteins as positive controls for the affinity purification reaction. IN = input, FT = flow through, WS = wash, AP = affinity pulldown precipitate.

Physical stripping of SAC proteins from metaphase kinetochores by cytoplasmic dynein is a major mode of SAC inactivation that precedes anaphase onset [22, 32, 33]. Defective stripping of SAC proteins leads to accumulation of these proteins at metaphase kinetochores [22, 32, 33]. We performed quantitative immunofluorescence of various SAC proteins at metaphase kinetochores using methods conceptually derived from the literature [34–37]. Cells treated with respective siRNAs for 48 hours were observed by time lapse imaging on gridded coverslips. Cells that achieved proper congression but arrested in metaphase for at least 80 minutes (indicating efficient LIC knockdown, see Fig 3B) were identified. The cover slips were co-immunostained for kinetochores (CREST), SAC proteins (Mad1/ Mad2/ Zw10/ BubR1) and chromosomes. The cells that had arrested for prolonged periods were located with the help of the etched grid and imaged by confocal immunofluorescence microscopy. Microscopic visualization demonstrated a clear retention of SAC protein signals at metaphase kinetochores in LIC2 depleted cells as compared to control cells (Fig 6), despite the prolonged duration of metaphase. Quantification of the individual SAC protein levels present on metaphase kinetochores revealed that all three attachment-sensing proteins tested, Mad1, Mad2 and Zw10, accumulate at metaphase kinetochores upon LIC2 depletion, strongly implicating LIC2 in stripping of these proteins from metaphase kinetochores (Fig 6A–6E). LIC2 stripped these SAC proteins as efficiently as LIC1 (Fig 6B, 6D and 6E). Strikingly, the ability of LIC2 to strip the tension-sensing BubR1 protein was pronounced and markedly higher than that of LIC1, which shows negligible ability to remove BubR1 (Fig 6F and 6G). In order to confirm that these SAC components physically bound to LIC2, we performed affinity purification of LIC2 from mitotic cryo-lysates of a cell line stably expressing LIC2-MTAP (LIC2 fused to a multifunctional C-terminal affinity purification tag; see Materials and Methods). We observed the presence of SAC components Mad1, Zw10 and BubR1 in the mitotic LIC2 affinity precipitates (Fig 7A). We could also observe robust pull down of other dynein subunits (Fig 7B), implying that LIC2-MTAP associated well with the dynein complex. However, none of these SAC components bound to mitotic lysates from a cell line stably expressing only the empty vector (Fig 7). These results implicate LIC2-dynein in a major new role in inactivating the tension-sensing arm of the SAC, in addition to its ability to inactivate the kinetochore-microtubule attachment-sensing arm in a manner similar to LIC1-dynein [22].

## Discussion

**Fig 8:**
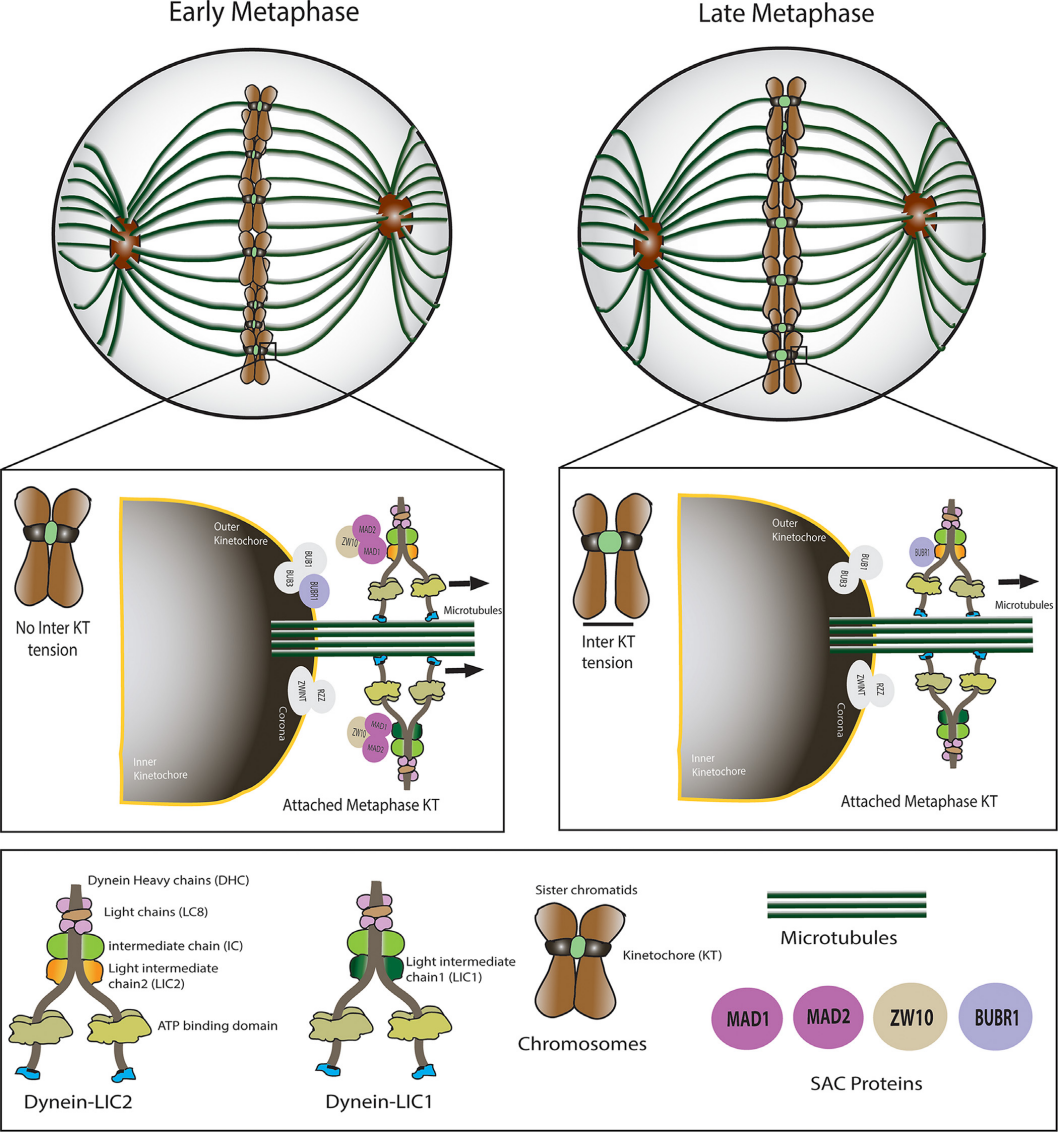
A model for key LIC2 functions in SAC silencing during mitosis. Novel functions of mitotic LIC2 uncovered from this study in silencing the spindle assembly checkpoint (SAC) are shown. LIC2-dynein strips attachment sensing SAC proteins (Mad1, Mad2, Zw10) from metaphase kinetochores, like LIC1-dynein. LIC2-dynein has the additional capability of causing removal of tension-sensing SAC protein BubR1, which is lacking in LIC1-dynein. The model offers a possible mechanistic explanation for the differential effects of the two LICs in mediating metaphase to anaphase progression.

The LIC2 subunit defines a separate sub-fraction of cytoplasmic dynein (LIC2-dynein) distinct from the one populated by LIC1 (LIC1-dynein) [17, 20, 21]. However, while LIC1-dynein has been shown to be required for metaphase to anaphase progression [22], the specific mitotic contributions of LIC2-dynein are unknown. We observed localization of LIC2 at mitotic kinetochores, also shown earlier [30], in addition to its earlier reported localization at spindle poles, the spindle microtubules and its midzone [25, 28, 30]. This observation, put together with several reports in the literature showing LICs as obligate members of the dynein complex [22, 23, 29], suggested that the LIC2-dynein sub-fraction performs important functions at mitotic kinetochores that had thus far not been characterized. The known role of LIC1-dynein in spindle assembly checkpoint inactivation prompted us to explore a possible role for LIC2-dynein in this function as well, given the high sequence identity of ~65% between both LICs. Our results have uncovered a novel function for LIC2-dynein during spindle assembly checkpoint inactivation and suggest key mechanisms that could explain these functions (Fig 8).

The metaphase arrest upon LIC2 depletion was observed to be as potent as that caused independently by LIC1 depletion (Figs 1–3). The siRNAs used in this study were specific only to their intended LICs and did not deplete the other LIC [S1 Fig, [22, 23]. The specificity of the LIC2 depletion phenotype was also established by the demonstration of functional rescue of human LIC2 depletion by a rat ortholog (Fig 2). These results showed that LIC2 has an equally strong and independent effect in ensuring metaphase to anaphase progression as LIC1. This idea is reinforced by the observation of an additive metaphase arrest phenotype upon co-treatment with the two siRNAs (Fig 2), but not upon increasing the dosage of either siRNA (not shown). We thus surmised that the mechanism of action of LIC2-dynein in ensuring metaphase to anaphase progression is at least partially distinct from that employed by LIC1-dynein. It is noteworthy that the prolonged metaphase phenotype penetrated to approximately 80% of the cell population after siRNA treatment, as evidenced by time-lapse imaging (Fig 3C). LIC2-dynein thus played a strong role in this phase of the mitotic cycle.

The strength of the LIC2 depletion mediated metaphase delay prompted us to explore the potential role of the spindle assembly checkpoint (SAC) in this phenotype, since the SAC is the main regulatory mechanism governing the timing of anaphase onset in eukaryotes [11, 22, 28]. The metaphase arrest indeed showed a strong dependence on the presence of a functional SAC (Fig 4), demonstrating the SAC as a major target of action of LIC2-dynein at metaphase. The involvement of the SAC was also corroborated by the high inter-kinetochore tension (reflected as increased inter-kinetochore distances) observed in LIC2-depleted metaphase cells (Fig 5). It is to be noted that these measurements were performed only with cells that had well congressed metaphase plates. The above results suggested that the SAC failed to be properly inactivated at metaphase kinetochores upon LIC2-depletion. The localization of LIC2 at kinetochores (Fig 1) substantiated our hypothesis. These results together suggested that LIC2-dynein could be involved in the removal of key SAC proteins from kinetochores, a function that has been well attributed to cytoplasmic dynein in the literature [22, 32–34, 36].

We therefore quantified the levels of SAC components at metaphase kinetochores upon LIC2 depletion, and compared with metaphase cells treated with control siRNAs using confocal immunofluorescence microscopy. It is noteworthy that we performed this assay only in cells that had spent abnormally prolonged periods in mitosis (confirmed by time-lapse imaging) upon LIC1/2 depletion (Fig 3), taking care also to quantify data only from well-congressed metaphase plates. Our results show that LIC2 depletion led to accumulation of kinetochore microtubule attachment-sensing SAC components Mad1, Mad2 and Zw10 at kinetochores. This function of LIC2-dynein appears similar the role of LIC1 dynein in stripping attachment sensors from kinetochores (Fig 6), [22]. Detailed quantitative analysis suggested that LIC1 and LIC2 remove Mad1 and Mad2 by similar mechanisms, since the magnitude of the individual LIC1/2 depletion phenotypes was comparable with that of co-depletion of both LICs (Fig 6). LIC2 also bound to the Mads and to Zw10 in mitotic lysates (Fig 7), supporting the inference that LIC2-dynein strips these proteins from mitotic kinetochores. It is possible however that there may be distinctions between the mechanisms employed by LIC1-dynein and LIC2-dynein in removing Zw10, since the co-depletion phenotype was approximately additive (Fig 6). This aspect of cytoplasmic dynein-dependent kinetochore Zw10 dynamics could be interesting to explore in the future.

The data that reveal the major mechanistic distinction between LIC1-dynein and LIC2-dynein pertain to their effects on the tension-sensing arm of the SAC, of which the SAC protein BubR1 is a major effector [32, 33, 38, 39]. As expected, LIC1 dynein did not appreciably affect levels of BubR1 at metaphase kinetochores (Fig 6F and 6G), [22]. The ability of LIC1-dynein to remove BubR1 was thus minimal. Unlike LIC1, LIC2 depletion led to a significant accumulation of BubR1 at metaphase kinetochores (Fig 6F and 6G), assigning a novel role in SAC silencing to LIC2-dynein. Recent reports had not attributed a role in SAC silencing to LIC2 [28, 29], based on qualitative microscopic visualization of SAC components at metaphase kinetochores. Our results, based on careful quantification of kinetochore SAC protein signals (which was missing in earlier reports) have revealed a clear contribution for LIC2 in stripping of SAC proteins. Quantification of SAC signals at kinetochores is an established method for determining SAC protein accumulation [22, 31, 32, 34, 35]. Accurate quantification is especially required for measuring kinetochore levels of BubR1, since it is not completely stripped off kinetochores even in anaphase, unlike the Mad proteins which are completely removed [36]. The role of cytoplasmic dynein in stripping of BubR1 from metaphase kinetochores has been debated [32–34, 40]. However, it was recently established that BubR1 is a bonafide cargo of cytoplasmic dynein [33]. Our results suggest that LIC2-dynein binds to BubR1 (Fig 7), and removes approximately 30% of kinetochore-localized metaphase BubR1 (Fig 6F and 6G) in Hela cells. LIC2 therefore plays a broader role in dynein mediated SAC silencing than LIC1 (Fig 6). A logical inference from this data is that LIC2 could act as a key molecular link that enables dynein mediated stripping of tension-sensing SAC proteins at metaphase, consistent with the known role of dynein in this function [22, 32–34, 36]. Indeed, recent studies suggest that the LICs could serve as key mediators of cargo binding specificity for the dynein motor [26, 27]. There however remains the possibility that LIC2-dynein could mediate kinetochore BubR1 removal by alternative mechanisms as well, which would be interesting to explore.

The differential SAC-stripping capabilities of LIC1 and LIC2 also explain their interesting mitotic arrest phenotypes. Depletion of either LIC1 or LIC2 individually from cells had the same apparent phenotype, a prolonged arrest in metaphase, both qualitatively and quantitatively (Fig 2). However, the additive magnitude of the phenotype seen upon LIC1 + LIC2 co-depletion (Fig 2A) strongly suggested at least partially distinct molecular mechanisms by which the two LICs facilitate progression through metaphase. Our SAC protein accumulation data elucidate this mechanistic distinction; LIC1 removes attachment-sensing SAC proteins, while only LIC2 can remove tension-sensing SAC proteins (Figs 6 and 8). There is a slight, possibly compensatory increase in LIC1 levels upon LIC2 depletion and vice versa (Fig 2B and 2E), which has also been reported earlier [41] We surmise that this increase would have some impact on the overall functional readout where the two LICs share overlapping functions and possibly common mechanisms, such as removal of attachment sensing proteins from kinetochores. However, such increases are unlikely to impact unique functions likely governed by distinct mechanisms, such as the removal of tension-sensing SAC proteins like BubR1. Our work suggests that these two activities of distinct dynein sub-populations collaborate to inactivate the SAC and facilitate the metaphase to anaphase transition. In the future, a deeper dissection of the molecular mechanisms governing these functional distinctions would significantly advance our understanding of mitotic progression. Such studies could also be potentially exploited to design therapeutic strategies for disorders related to cell division.

## Materials and Methods

### Cell culture and cell synchronization

HeLa (cervical cancer cell line) was obtained from the ECACC. HeLa cells were grown in Dulbecco's Modified Eagle Medium (DMEM-high glucose). The H2B-mCherry-GFP-α tubulin line (gift from Daniel Gerlich) was grown in medium supplemented with hygromycin B and puromycin. A cell line stably expressing human LIC2-MTAP-MVenus (hLIC2-MTAP) was generated in U2OS cells (human bone osteosarcoma epithelial cell line). The hLIC2 was cloned in to MTAP-mVenus multifunctional vector, which consists of tandem Flag-Streptavidin binding protein (SBP)-Histidine tags for affinity purification and yellow fluorescent protein (YFP) for visualization [42]. The MTAP_mVenus_LIC2 cells were grown in medium supplemented with hygromycin B. Cells were visualized in a phase contrast inverted microscope (Nikon Eclipse TS 100). The fluorescent tag expressing stable cell line was visualized on fluorescence microscope (Nikon) as well as Leica TCS SP5 II and Leica TCS SP8 confocal microscopes. To prepare cryo-lysates, cells were treated with nocodazole (400 nM) and subsequently released from the drug by washing with PBS for 1 hour. Mitotic cells were harvested, flash frozen and physically ground under cryogenic conditions to obtain cryo-grindates (cryogenic lysates) as reported earlier [43, 44]. Nocodazole was used for enriching mitotic cells for biochemical experiments only.

### Plasmid constructs, siRNAs and transfection

Rat LIC2 cDNA was cloned into the pCMV 3Tag 3B vector (Agilent Technologies) and sequenced. The transfection of this plasmid was performed using Lipofectamine 2000 transfection reagent (Invitrogen) as described in the manual. SiRNAs against different human genes were procured from Dharmacon. The following sequences and working concentrations were used: GFP (negative control): CAU GAA GCA GCA CGA CUU C (100nM), Mad2: GAG UCG GGA CCA CAG UUU (100nM), BubR1: GGA GAU CCU CUA CAA AGG GUU (100nM), LIC1: GAA AGU UUG UAC AUG AGA A (100nM), LIC2a: ACC UCG ACU UGU UGU AUA A (100nM), LIC2b: GCC GGA AGA UGC AUA UGA A (100nM). All the siRNA sequences have been previously used and published by different groups [22, 23]. These individual siRNAs were transfected using reagent Dharmafect 1 (Dharmacon/ Thermo Scientific) for 48 hours. During co-depletion of LIC1 + 2, 100 nM each of the two siRNAs were used. For rescue experiments in cell lines, plasmids were transfected on day 1 followed by siRNA transfection on day 2 and observation at 48hrs after siRNA transfection. Metaphase index was calculated by counting metaphase cells as a fraction of total living cells under a fluorescence microscope to visualize chromosomes, which were stained by 4', 6-diamidino-2-phenylindole (DAPI) or Syto 13 (Invitrogen).

### Immunoblotting

Cell lysates were prepared by directly adding Laemmli buffer (SDS PAGE loading dye) into the culture plate, harvesting the cells and heating the sample at 95°C for 5 min. Samples were resolved on SDS-PAGE, followed by transfer of proteins on to polyvinylidene difluoride (PVDF) membrane (Millipore). Blots were blocked with 5% skimmed milk followed by incubation in primary antibody for 1 hr at room temperature or at 4°C overnight, washed and incubated with secondary HRP conjugated antibodies for 1 hr at room temperature, and washed extensively. The antibody dilutions used for immunoblotting were: LIC1–1:500, LIC2–1:500, Mad1–1:500, Mad2–1:250, β-actin- 1:2000, IC74–1:1000, myc—1:2000, anti-mouse HRP 1:10000, anti-rabbit HRP- 1:10000. The chemiluminescence signal was developed using the Luminata Forte reagent (Millipore) and captured in the Image Quant 4000 (GE).

### Affinity precipitation

A Streptactin-HP (GE) affinity column was used for purification of the MTAP-mVenus-hLIC2 protein from the stably expressing cell line, using the Streptavidin-binding-protein tag imparted by the MTAP-mVenus vector [42]. Cell lysates were prepared by cryogenic grinding for 1 hour and incubated for at least 20 minutes on ice in lysis buffer containing 50 mM Tris pH 7.5, 125mM sodium chloride, 1mM EGTA, 0.2 NP-40, 5% glycerol, protease inhibitors and phosphatase inhibitors (Roche/ Pierce). The cell lysates were loaded with flow rate of 0.5ml/min on Streptactin-HP affinity column. The flow through was collected and the column washed with wash buffer containing 50 mM Tris pH 7.5, 250 mM sodium chloride, 0.2% NP-40 (Nonidet P40), protease inhibitors and phosphatase inhibitors. Finally, the purified MTAP_LIC2 was eluted with elution buffer containing 25 mM Tris pH 7.5, 125 mM sodium chloride, 2.5mM D-Biotin along with protease inhibitors and phosphatase inhibitors. Purified samples were analyzed by SDS PAGE resolution followed by immunoblotting.

### Antibodies

The following primary antibodies against respective antigens were used: LIC1, Mad1, Mad2, Zw10 (Pierce/ Thermo-scientific); LIC2 (Pierce/ Thermo-Scientific PA5-25392 for immunoblotting and immunofluorescence staining, Abcam ab178702 for immunoblotting), BubR1 (Bethyl laboratories); dynein heavy chain from Abcam and ProteinTech; α-tubulin (Dm1α), β–actin, monoclonal antibodies from Sigma; IC-74 monoclonal antibody from Abcam. The CREST antibody was purchased from Antibodies Incorporated. HRP conjugated anti-mouse and anti-rabbit secondary antibodies were purchased from Sigma for Western blotting. The fluorophore attached secondary antibodies for immunofluorescence analyses were purchased from Jackson Immunoresearch.

### Immunofluorescence staining

HeLa cells were grown on glass coverslips, washed with PBS, and fixed in 4% formaldehyde or chilled methanol. Fixed coverslips were incubated with blocking buffer (PBS + 1% bovine serum albumin + 0.5% Triton X-100) for 1 hour, followed by incubation for 1 hour with primary antibody, washed and incubated with secondary antibodies in a humidified chamber. The following antibody dilutions were used for immunofluorescence staining: LIC1–1:250, LIC2–1:50, Mad1–1:100, Mad2–1:100, Zw10–1:100, BubR1–1:500, IC74–1:500, dynein heavy chain—1:200, α-tubulin—1:1000, CREST—1:50. DAPI staining was performed (1:10,000 concentration of a 5 mg/ ml stock solution) for 1 minute, cells washed in PBS and water, and mounted on a glass slide using mounting medium (Prolong Gold, Invitrogen). Imaging of fixed coverslips was performed after drying the mounting medium for about 12 hours after mounting.

### Microscopy

#### Immunofluorescence imaging of cells

Fixed cell image acquisition was performed on a Leica TCS SP5 II or Leica TCS SP8 laser scanning optical confocal microscope using a HCX PL APO CS 63X- 1.4 numerical aperture oil immersion objective. All acquisition settings were kept identical for control as well as test samples. Only well-congressed metaphase cells were imaged to analyze SAC protein accumulation at kinetochores.

#### Time-lapse imaging

HeLa cells were grown on coverslips and suitably treated. The coverslips were placed on custom-designed aluminium slide containing chambers. The autoclaved coverslips were affixed at one side of the chamber using VALAP (mixture of paraffin, Vaseline and Lanolin in 1:1:1 ratio). Conditioned media was filled into the well thus formed. The other side of the well was closed by the coverslips on which cells were growing using autoclaved silicone grease. Time-lapse imaging was performed for 12 hours with a 2 minutes time interval between two frames with four different positions on each coverslip in bright field mode of motorized microscope. In some experiments, Labtek chambered cover slips (Nunc) were used for time lapse imaging.

#### Gridded coverslip experiments

HeLa cells were grown on the gridded coverslips (Electron Microscopy Sciences), after 36 hours of siRNA transfection the coverslips were placed on the aluminium slide as described above followed by time lapse imaging for 12 hours with a 2.5 minutes time interval between two frames, with four different positions on each coverslip in bright field mode of the motorized microscope. The same gridded coverslips were fixed and used for immunostaining. The grids on the coverslips were used to locate the very same cells that got arrested in mitosis for prolonged periods (over 1.5 to hours); only these cells were used for further fixed cell imaging following immunostaining.

#### Image analysis

Fluorescence image analysis was performed on the Imaris software suite (version 5.7, Bitplane), Leica offline image analysis software (LAS) and ImageJ.

#### Checkpoint protein quantification at kinetochores

We imaged control metaphase cells and for LIC1/LIC2/LIC1 + 2 depleted cells, only the cells that had properly congressed metaphase plates and had arrested in metaphase for more than 1.5 hrs, which we located from time lapse imaging with the help of the grid etched on the coverslip. The metaphase cells were stained for checkpoint proteins (Mad1/ Mad2/ Zw10/ BubR1), kinetochore (CREST), DNA (DAPI) and microtubules (α-tubulin). For Mad1, Mad2 and Zw10, the 20 visibly brightest kinetochores were analyzed, while all kinetochores were taken for analysis of BubR1 levels. Mad1, Mad2 and Zw10 are the attachment sensing SAC proteins that get almost completely removed in late metaphase/ anaphase from kinetochores [11, 34, 36]. Therefore, visible retention of these SAC proteins on aligned metaphase kinetochores indicates metaphase arrest due to failure of SAC inactivation. This method of quantifying the brightest kinetochores has been reported in the literature [22]. However BubR1, a tension sensing SAC protein, is partially retained on kinetochores even in anaphase [11, 34, 36]. In order to detect even small changes in BubR1 levels dependent on LIC2-dynein that may not be visually obvious, we quantified all kinetochores. The images were reconstructed from z-stacks for 3-dimensional visualization in the Imaris suite (Bitplane). The integrated fluorescence intensities of various checkpoint proteins at kinetochores in metaphase cells were quantified using Imaris. We made a sphere of diameter 0.45 μm (average kinetochore size of HeLa cells) around each kinetochore and measured the intensity of SAC proteins on the sphere (kinetochore). We normalized the intensity of SAC proteins to the intensity of kinetochores (CREST) from the same sphere and plotted the ratio in scatter plots using the Graphpad PRISM software. Local background was subtracted from all intensity measurements using features of the Imaris software. Three-dimensional quantification has been used earlier to measure kinetochore SAC protein levels in mammalian cells [35]. Our method detailed above gave us comparable fold differences for normalized SAC protein intensities with the original and well accepted method published for kinetochore SAC protein quantification [22, 32, 34].

### Statistical analysis

The number of cells counted per experiment for statistical analysis is mentioned in respective figure legends. The graphs in all figures are depicted with error bars, mean of at least 3 experiments +/− SD or SEM. Statistical significance was calculated by student t-test or one way ANOVA with Tukeys comparison method. Scale bars (μM) for images are indicated in the respective legends. Graphs were created in Graph Pad PRISM software.

## Supporting Information

S1 Fig Nucleotide BLAST results showing mismatches in sequence alignment of both LIC2 specific siRNAs (LIC2a and LIC2b) with the rat LIC2 sequence. (TIFF)

S1 Movie Fluorescence time-lapse movie of control live H2B-mCherry GFP-α-tubulin HeLa cell showing the timing from NEB (nuclear envelope breakdown) to anaphase onset. Green indicates microtubules and red indicates metaphase plates (chromosomes). Time-lapse imaging was performed for 12 hours with a 2-minute time interval between frames at four different stage positions on a motorized confocal microscope.
(MOV)

S2 Movie Fluorescence time-lapse movie of LIC2 depleted live H2B-mCherry GFP-α-tubulin HeLa cell showing the timing from NEB (nuclear envelope breakdown) to anaphase onset (metaphase arrest > 80minutes). Green indicates microtubules and red indicates metaphase plates (chromosomes). Time- lapse imaging was performed for 12 hours with a 2-minute time interval between frames at four different stage positions on a motorized confocal microscope.
(MOV)

S3 Movie Fluorescence time-lapse movie of LIC2 depleted live H2B-mCherry GFP-α-tubulin HeLa cell showing prolonged arrest in mitosis (> 4 hours) followed by death. Green indicates microtubules and red indicates metaphase plates (chromosomes). Time-lapse imaging was performed for 12 hours with a 2-minute time interval between frames at four different stage positions on a motorized confocal microscope.
(MOV)

S4 Movie Fluorescence time-lapse movie of LIC1+2 depleted live H2B-mCherry GFP-α-tubulin HeLa cell showing prolonged metaphase arrest. Green indicates microtubules and red indicates metaphase plates (chromosomes). Time-lapse imaging was performed for 12 hours with a 2-minute time interval between frames at four different stage positions on a motorized confocal microscope.
(MOV)
