# Supplementary figures and images for: Dynein Light Intermediate Chain 2 Facilitates the Metaphase to Anaphase Transition by Inactivating the Spindle Assembly Checkpoint
Source: PLoS One. 2016 Jul 21;11(7):e0159646. doi: 10.1371/journal.pone.0159646 (PMC4956306; doi:10.1371/journal.pone.0159646)

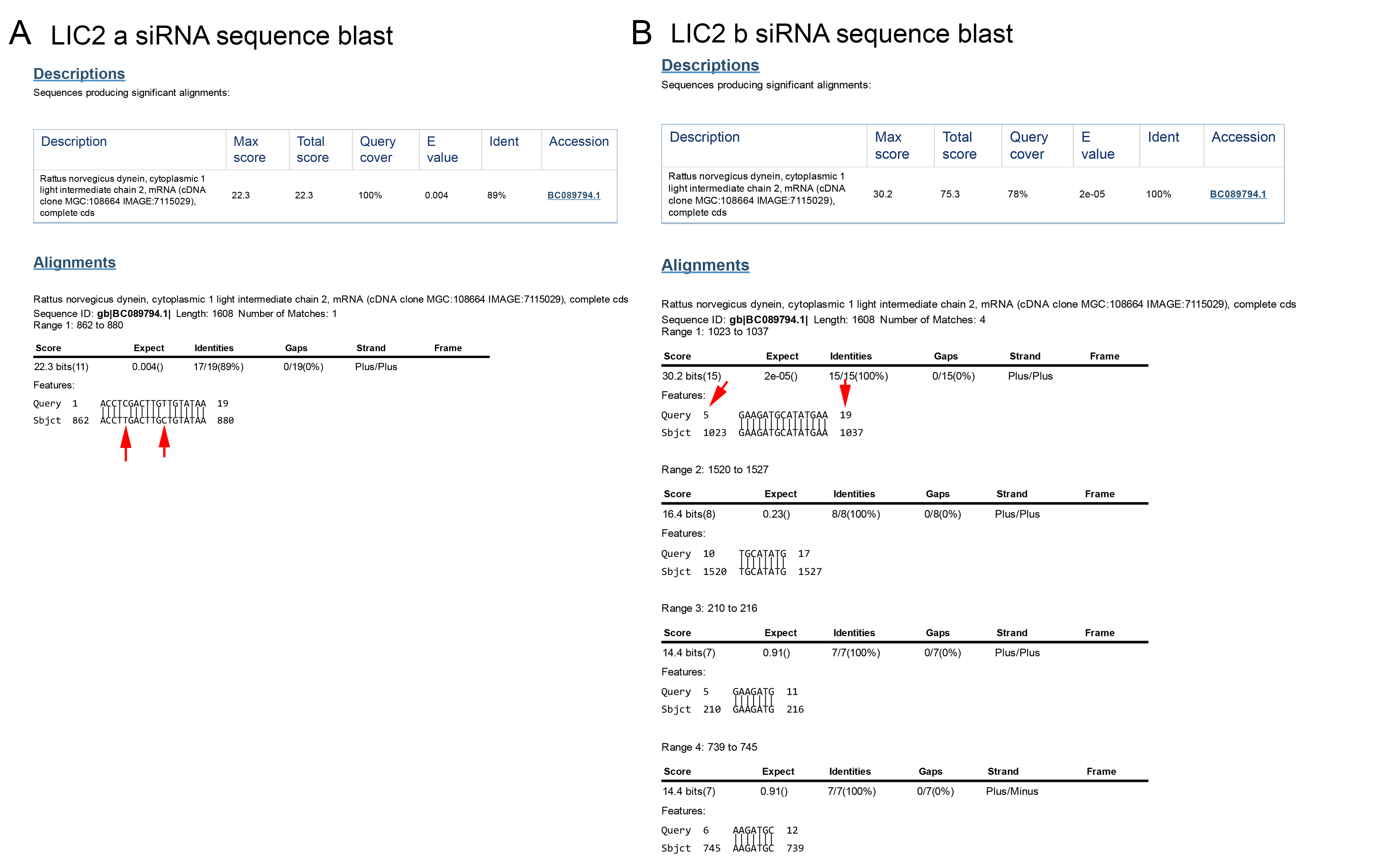

Supplement: S1 Fig — (TIFF) [file pone.0159646.s001.tiff]
